# How attention factors into executive function in preschool children

**DOI:** 10.3389/fpsyg.2023.1146101

**Published:** 2023-07-12

**Authors:** Aditi V. Deodhar, Bennett I. Bertenthal

**Affiliations:** ^1^HANDS in Autism^®^ Interdisciplinary Training and Resource Center, Riley Hospital for Children at IU Health, Department of Psychiatry, IU School of Medicine, Indianapolis, IN, United States; ^2^Developmental Cognitive Neuroscience Lab, Department of Psychological and Brain Sciences, Indiana University-Bloomington, Bloomington, IN, United States

**Keywords:** executive function, attention, attentional control, preschool development, confirmatory factor analysis, factor structure

## Abstract

Executive Function consists of self-regulation processes which underlie our ability to plan, coordinate, and complete goal-directed actions in our daily lives. While attention is widely considered to be central to the emergence and development of executive function during early childhood, it is not clear if it is integral or separable from other executive function processes. Previous studies have not addressed this question satisfactorily because executive function and attention are multidimensional constructs, but they are often studied without differentiating the specific processes that are tested. Moreover, some studies consist of only one task per process, making it difficult to ascertain if the pattern of results is attributable to different processes or more simply to task variance. The main aim of this study was to more fully investigate how attention factored into the underlying structure of executive function in preschool children. Preschool children (*n* = 137) completed a battery of tasks which included executive function (i.e., response inhibition, working memory) and attentional control (i.e., sustained attention, selective attention) processes; there were two tasks per process. Confirmatory factor analyses (CFA) were conducted to test which of three models fit the data best: (1) a unitary one-factor model with attention loading onto the same factor as other executive function processes, (2) a two-factor model with attention loading onto a separate factor than other executive function processes, or (3) a three-factor model with attention, response inhibition, and working memory as separate factors. Fit indices and model comparisons indicated that the two-factor model fit the data best, suggesting that attentional control and executive function were related, but separable. Although this study is not the first to advocate for a two-factor model during the preschool years, it is the first to suggest that the two factors are attentional control and executive function, not working memory and response inhibition. One important implication of these findings is that a complete assessment of executive function during the preschool years necessitates measuring not only response inhibition and working memory, but attentional control as well.

## 1. Introduction

Executive Function (EF) refers to self-regulation processes which underlie our ability to plan, coordinate, and complete goal-directed actions in our daily lives. EF emerges during infancy and undergoes substantial development during the preschool years (Diamond, 2013; Griffin et al., 2016). EF is considered foundational to development since early individual differences are predictive of later cognitive/academic performance (e.g., Fitzpatrick and Pagani, 2012) as well as successful social interactions (e.g., de Wilde et al., 2016). Deficits in EF are associated with developmental disorders including Autism Spectrum Disorder (ASD) and Attention-Deficit/Hyper-Activity (ADHD) (Ruff and Rothbart, 2001; Griffin et al., 2016). By now there has been a good deal of research examining how EF quantitatively and qualitatively changes as a function of age, with much consideration given to how best to conceptualize the structure of EF throughout childhood. While EF consists of multiple related dimensions in older children and adults (Miyake et al., 2000; Lehto et al., 2003), it is still not clear if EF is best conceptualized as a multi-dimensional or a unitary construct during the preschool years (Lerner and Lonigan, 2014; Nelson et al., 2016).

Attention is widely considered the process common to all EF subdomains, regardless of how the EF structure itself is conceptualized (Kane and Engle, 2003; Posner and Rothbart, 2007; Garon et al., 2008), It is well established that attention plays a central role in EF development during the preschool years (Garon et al., 2008). Consistent with this idea, previous studies demonstrate that facilitating children’s attention by increasing the number of stimulus cues or their duration improves children’s performance on EF tasks (e.g., Kirkham et al., 2003; Bertrand and Camos, 2015). Yet, studying how attention relates to EF in this manner does not directly address if children’s attention is separate from EF or if their attention should be viewed as integral to EF. Put another way, these studies do not indicate if Attentional Control (AC) is an independent and separable factor from EF or if attention is integrated with each dimension of EF in preschool children. The main limitation in previous studies of attention is that authors often overlook the fact that attention is not a monolithic construct; AC involves multiple processes, including sustained and selective attention (Fan et al., 2002; Posner, 2012). The main aim of the current study is to examine the underlying latent structure of EF with the inclusion of tasks directly assessing sustained and selective attention in preschool children.

Executive Function consists of three related but distinct subdomains: response inhibition (i.e., inhibition of a prepotent or automatic response in order to make a target response), working memory (i.e., maintenance and manipulation of information for a short period of time), and set shifting (i.e., flexible shifting from one task to another) in adults and older children (Miyake et al., 2000; Lehto et al., 2003; Garon et al., 2008). It is not clear if this pattern extends to preschool children. The prevailing view is that EF is an undifferentiated construct during the preschool years which only differentiates into the subdomains later in childhood (Nelson et al., 2016). Consistent with this view, several studies report that response inhibition and working memory are highly correlated and the tasks used for measuring these subdomains load onto a single factor when assessed via factor analysis (Hughes and Ensor, 2007; Wiebe et al., 2008, 2011; Hughes et al., 2010; Welsh et al., 2010; Willoughby et al., 2010; Visu-Petra et al., 2012; Nelson et al., 2016). The competing view suggests that EF subdomains are related, but still distinct in preschool children and exhibit different developmental trajectories throughout childhood (Zelazo and Carlson, 2012). Consistent with this view, several studies report that the tasks associated with response inhibition and working memory load onto separate factors (González Osornio and Ostrosky, 2012; Miller et al., 2012; Schoemaker et al., 2012; Lerner and Lonigan, 2014).

An additional complication in conceptualizing the early structure of EF arises from different forms of response inhibition. This component is measured in both “hot” or emotionally laden contexts where there is the presence of salient rewards or punishments, as well as in “cool” or emotionally neutral contexts (Carlson, 2005). The evidence is inconclusive with regard to whether these two forms of response inhibition reflect a unitary factor (Sulik et al., 2010; Allan and Lonigan, 2011) or distinct factors (Carlson et al., 2002; Willoughby et al., 2011). In summary, the extant literature provides conflicting results regarding whether EF is a unitary vs. multi-dimensional construct in preschool children.

Some common explanations for these different conclusions are related to “task impurity” and task differences between studies. “Task impurity” refers to the fact that performance on EF tasks is not only based on the purported EF subdomain, but other self-regulation processes or nonexecutive skills (Nelson et al., 2016). For example, children’s performance on Stroop-like tasks, which require children to respond to a stimulus (e.g., sun) with a counterintuitive response (e.g., night), are not only dependent on the their ability to inhibit the automatic response (e.g., sun goes with day), but also their ability to pay attention to the new stimulus–response pairing in working memory (Wiebe et al., 2008). It is also difficult to ascertain how other task factors such as stimulus salience or response modality may influence task performance (Miyake et al., 2000; Miller et al., 2012). These considerations are most evident in studies which use only one task/measure to assess an EF subdomain. In these cases, it is difficult to know if the observed pattern of associations reflects the underlying structure or something more idiosyncratic to the specific tasks. This ambiguity is especially troubling when the study is designed to test how different subdomains are related (Lerner and Lonigan, 2014). One solution is to include multiple tasks/measures per subdomain and then pool the common variance among the tasks/measures via composite scores or factor analysis for a “purer” assessment of the subdomain (Miyake et al., 2000; Wiebe et al., 2011).

Attention is widely viewed as pivotal to a central executive (Baddeley, 2002; Kane and Engle, 2003) and is considered foundational to the development of EF subdomains (Garon et al., 2008). For example, selecting and sustaining attention toward relevant information and inhibiting irrelevant information narrows focus and creates an “attentional spotlight,” as well as enhances the maintenance and processing of relevant information in working memory, which has a limited capacity (Gathercole et al., 2008; Posner and Fan, 2008). This close relationship between working memory and sustained and selective attention is illustrated in studies which reveal that preschool children with lower working memory capacity perform worse on selective attention tasks (Espy and Bull, 2005) and are more likely to exhibit attention problems in the classroom (Gathercole et al., 2008). In addition, response inhibition is critical for a child to successfully select and sustain attention on various problem solving tasks, such as completing a puzzle (Allan et al., 2015). More generally, children who perform better on response inhibition tasks also tend to perform better on sustained attention tasks (Reck and Hund, 2011). While these examples suggest some functional relation between AC and specific EF subdomains in preschool children, they neither confirm nor deny whether AC fits into the underlying structure of EF. Critically, these studies are limited to single measures of AC and EF, and thus it is difficult to ascertain whether the reported covariations are a function of AC and EF or merely a function of some other variable common to both processes.

Studies that do include AC and multiple EF subdomains are riddled with a number of confusions and inconsistencies. For instance, Veer et al. (2017) found that children with better selective attention exhibited superior working memory and response inhibition concurrently and 6 months later. Nevertheless, other studies indicate that the relation between attention and different EF subdomains may not be so straightforward. For example, Lan et al. (2011) tested how US and Chinese preschool children’s working memory and response inhibition related to their performance on a visual search task. The children’s working memory was related to visual search performance in both countries, but response inhibition was related to visual search performance only in China. Similarly, Lin et al. (2019) found that performance on a sustained attention task (Continuous Performance Task-CPT), was significantly correlated with one “hot” EF task, but was only marginally correlated to a second “hot” EF task as well as to the “cool” EF tasks. It is not clear if these inconsistent results are an artifact of “task impurity” or task selection (Miller et al., 2012), or if they signify true separability between attention and EF in preschool children. This ambiguity may again result from study designs including only one measure per subdomain, making it difficult to know if children’s task performance reflects their attention and EF, or something more specific to the task, such as stimulus salience or domain knowledge (e.g., Griffin et al., 2016).

There have been several calls to design studies that include multiple measures per subdomain to help ensure that studies are truly assessing the desired subdomain (Veer et al., 2017; Lin et al., 2019). Allan et al. (2015) examined how working memory, response inhibition, and sustained attention were related by having three measures per subdomain in a preschool sample. They found that EF tasks (working memory and response inhibition) loaded onto a different factor than sustained attention, suggesting some distinction between EF and AC in preschool children. Critically, however, Allan et al. (2015) focused exclusively on sustained attention and did not include any assessment of selective attention. Thus, even this more comprehensive study treated attention as a unitary construct, limiting our knowledge of how attention may fit within the EF structure.

Even though many researchers have postulated that attention regulates EF (Awh et al., 2006; Garon et al., 2008), the tendency to assess AC as a monolithic construct limits our knowledge of how AC relates to EF. One notable exception is the work by Posner, Rothbart and colleagues who postulate that different components of AC are associated with an attention network that develops gradually and leads to EF changes in early childhood (Rueda et al., 2005; Posner and Rothbart, 2007). Posner’s Attention Network Theory (Posner, 2012) indicates that AC consists of three related but distinct subdomains in adults: sustained attention (i.e., maintenance of a narrow focus on a single object or event for a prolonged period of time), selective attention (i.e., disengagement from one target in order to orient toward another target), and executive attention (i.e., monitoring and resolving conflicting information). To more fully address how AC factors into the latent EF structure, our study was informed by this theory and included measures of both sustained and selective attention (see discussion for reasons that executive attention was not included).

While attention is often considered a central process in most theories of EF during early childhood, there are very few studies directly assessing different AC processes and testing how they contribute to the underlying structure of EF. For quite some time there has been a debate in the literature regarding whether executive function is structurally consistent with one or two factors during the preschool years (Griffin et al., 2016). Based on our review of the literature, we believe that this debate is somewhat misguided. The issue is not whether working memory and response inhibition are separable subdomains, but rather whether attentional control and executive function are separable subdomains. Critically, studies suggesting that executive function during the preschool years consists of two separable factors may have confounded measures of attentional control with measures of either working memory or response inhibition. For example, Lerner and Lonigan (2014) reported that response inhibition and working memory were distinct factors, but the tasks assessing response inhibition for the most part also involved greater attentional demands than the working memory tasks. As such, the response inhibition factor was confounded with children’s attentional control. If this hypothesis is correct, then we should be able to demonstrate that the tasks used for measuring executive function are best explained in terms of two different factors, but not the two factors that have been previously suggested. Instead, we predict that one factor involves attentional control (selective and sustained attention) and the other factor involves working memory and response inhibition. Converging support for this hypothesis would be provided if we are able to demonstrate that the two subdomains of working memory and response inhibition are not differentiable during the preschool years.

The main objective of the current study was to test how AC and EF were related in preschool children between 3.5 and 5 years of age. Specifically, we sought to identify the underlying structure of children’s EF when including measures to also assess both sustained and selective attention. To this end, preschool children completed a battery of tasks associated with EF subdomains (i.e., response inhibition in “cool” and “hot” settings, working memory) and AC processes (i.e., sustained attention, selective attention). Development of the study design was based on a careful review of the literature and extensive pilot testing to ensure that each subdomain had more than one measure that was applicable to the entire age range while ensuring considerable variability in children’s performance.

Confirmatory Factor Analyses were conducted to examine the underlying structure in the current battery of EF and AC measures. The main advantage of CFA over similar analytic techniques such as Exploratory Factor Analysis (EFA) and Principal Component Analysis (PCA) is that this method enables researchers to test pre-specified latent structures based on theory and prior empirical studies (Nelson et al., 2016; but see Hurley et al., 1997; Prudon, 2015). Further, CFAs allow for model comparison that directly test which of two or more competing models fit the data better. The utilization of CFAs has steadily increased as more empirical studies investigate the underlying EF structure at different stages throughout childhood (e.g., Lehto et al., 2003; Wiebe et al., 2011; Miller et al., 2012; Lerner and Lonigan, 2014), allowing for increasingly more specific investigations and inferences about how EF structure changes throughout childhood. The current study was designed to add new insights into how sustained and selective attention may influence this EF structure in preschool children.

CFAs were conducted to test whether a one-factor model with all EF and AC measures loaded onto the same factor fit the data better than a two-factor model with all EF measures associated with one factor and AC measures associated with a second factor. We predicted that the two-factor model would fit the data better than the one-factor model, aligning with Allan et al. (2015) and suggesting that AC is separable from EF. Since there is some evidence in the literature that EF subdomains (e.g., response inhibition, working memory) might be separable in preschool children, an additional three-factor CFA was conducted with response inhibition and working memory as separate factors to see if this model fit the data better than the one-or two-factor models. Given our hypothesis that previous studies (e.g., Lerner and Lonigan, 2014) reporting working memory and response inhibition as separable factors was due to failing to control for the confound between these two processes and attentional control, we predicted that the two-factor model combining response inhibition and working memory as one factor and attentional control as the second factor would be preferable to the three-factor model.

## 2. Method

### 2.1. Participants

One hundred and thirty-seven preschool children (69 female, M = 50.79 months, range = 41–60 months) participated in the study; see Table 1 for number of participants per age range. The majority of children participating in this study were Caucasian (83.94%), and the remainder were either Asian-American (13.14%) or African-American (2.92%). Parents reported their education level on a seven-point scale: 1 = did not complete high school, 4 = Associate’s degree or equivalent 2 year undergraduate degree, 7 = completed a graduate degree (M = 5.73, range = 2–7). All participants lived in a university town in the Midwest. They were recruited from families who had previously participated in developmental research studies in the department, expressed interest at local community events, or from word of mouth at preschools and local activities. All children included in the study had no history of developmental delays (e.g., language delay) or other significant medical issues (e.g., hearing or visual difficulties, ASD, relative with ASD) based on parental report. The study protocols were reviewed and approved by the Indiana University Institutional Review Board (IRB). Parents provided written informed consent before the start of the study session to participate in this study.

**Table 1:** Number of participants per age range.

Age range	Female	Male	Total
41–48 months	24	25	49
48–54 months	27	20	47
54–60 months	18	23	41
Total	69	68	137

### 2.2. Procedure

Children participated in one lab session lasting between 50 and 65 min. In order to keep children engaged and motivated, they were shown a piece of paper with a snowman who needed to retrieve his hat 10 paces away; each pace was demarcated by a snowflake. Children were told that they could help the snowman get one step closer to the hat with every task completed; they were reminded to color in a snowflake after the completion of every task. All children completed tasks in the same order: low-frequency continuous performance task, spin the pots, visual search task, circle/triangle, high-frequency continuous performance task, digit span, flanker task, and wrapped gift; see below for task descriptions. Participants were also allowed bathroom or snack/water breaks between tasks as needed. All testing sessions were conducted in a single room and were video recorded for offline scoring.

### 2.3. Executive function tasks

#### 2.3.1. Circle/triangle

The circle/triangle task was based on the day/night task developed by Gerstadt et al. (1994) to assess children’s response inhibition in a “cool” context. In this task, the experimenter showed the child a picture of a circle and a triangle and asked the child to label each shape. The experimenter then introduced a “silly game” and instructed the child to say “triangle” when he saw a picture of a circle and “circle” when he saw a picture of a triangle. The pictures were presented in an ABBABAAB order to ensure that they did not consistently alternate, and no picture was presented more than twice in a row; there were a total of 16 trials. The outcome measure was the proportion of trials where the child’s first response was correct. Cohen’s kappa was 0.87 between two scorers for 105 participants. It is important to note that similar tasks are used to assess executive attention (e.g., Steele et al., 2012), and thus could also be considered a measure of attentional control.

#### 2.3.2. Wrapped gift

The wrapped gift task was adapted from Kochanska et al. (2000) to assess response inhibition in a “hot” context. The child was presented with a gift bag and was told there was an exciting prize inside. The experimenter told the child she needed to get tissue paper to make the gift bag ready and instructed the child not to touch or peek inside the gift bag until she returned. The experimenter left the testing room and returned with the tissue paper after 4 min had elapsed. The outcome measure was a composite of latency to touch the bag and latency to look inside it. Cohen’s kappa was 0.94 for latency to first touch and 0.96 for latency to first peek between two scorers for 105 participants. If the bag was not touched or looked into, children received a maximum score of 480 (corresponding to the sum of the total number of elapsed seconds for both measures).

#### 2.3.3. Spin the pots

The spin the pots task was adapted from Hughes and Ensor (2005) and assessed children’s working memory for visual–spatial information. A rubber ducky was hidden under one of eight distinctly colored cups turned upside down and arranged in a circle on a lazy Susan tray. The experimenter then occluded the hiding locations from the child’s view and spun the lazy Susan so that each cup was in a new location relative to the child. The child was then instructed to find the hidden rubber ducky. Each trial ended when the child found the rubber ducky or failed to find the rubber ducky after three attempts. There were eight trials in which each colored cup was the hiding location for one trial. The outcome measure was the proportion of correct trials in which the child found the rubber ducky on the first search attempt. Cohen’s kappa was 0.98 between two raters for 104 participants.

#### 2.3.4. Digit span

The digit span task was adapted from Davis and Pratt (1995) and assessed children’s working memory for verbal information. On each trial, the child listened to a one-to-seven-digit sequence and was asked to repeat it. There were three trials per digit sequence length, and trials progressed in a *n* + 1 order (i.e., three trials for one-digit sequences, three trials for two-digit sequences, etc.). The task stopped when the child responded incorrectly on two of the three trials as it was assumed the child would respond incorrectly on the remaining trials with longer digit spans. The outcome measure was the proportion of trials with correct responses out of the total number of trials that could have been administered. Cohen’s kappa was 0.97 between two raters for 101 participants.

### 2.4. Attentional control tasks

#### 2.4.1. Low-frequency continuous performance task

The low-frequency continuous performance task was adapted from Corkum et al. (1995) and assessed children’s sustained attention. The child saw a sequence of animals (i.e., cat, alligator, dog, pig, or elephant) on an iPad or touchscreen laptop using the Paradigm Experimenter software (Perception Research Systems, Walnut Creek, California). The child was instructed to touch the screen whenever he saw a cat and not touch the screen whenever he saw any other animal. Each animal was presented for 1,200 milliseconds (ms) and each inter-trial interval (ITI) was 750 ms. There were 100 trials with a cat presented on 20% of the trials; the entire task lasted approximately 4 min. The outcome measure was d-prime to control for response biases (Macmillan and Creelman, 2005). It was calculated by subtracting the z-transformed false alarm rate (i.e., the proportion of trials on which the child touched the screen when an animal besides the cat was present) from the *z*-transformed hit rate (i.e., the proportion of trials on which the child touched the screen when the cat was present).

#### 2.4.2. High-frequency continuous performance task

The high-frequency continuous performance task was adapted from Rezazadeh et al. (2011) and assessed children’s sustained attention. The child saw a sequence of common modes of transportation (i.e., car, school bus, boat, plane, and train) on an iPad or a touch-screen laptop controlled with the Paradigm Experimenter software. Children were instructed to touch the screen whenever they saw one of the target stimuli (i.e., all modes of transportation but the car) and not touch the screen whenever they saw the distractor stimuli (i.e., the car). Each mode of transportation was presented for 1,200 ms and each ITI was 750 ms. There were 100 trials; a target was presented on 80% of the trials and the car was presented on 20% of the trials; the entire task lasted approximately 4 min. The outcome measure was d-prime.

#### 2.4.3. Visual search task

The visual search task was adapted from Breckenridge et al. (2013) and assessed children’s selective attention. The child saw an array of twenty green apples and twenty red strawberries on an iPad or a touchscreen laptop controlled with the Paradigm Experimenter software. Each array also included one randomly placed red apple, and the child was instructed to find and touch the red apple on each trial. There were 32 trials and each trial ended when the child found the red apple or 10 sec had elapsed; the ITI was 3 sec. The outcome measures were accuracy and reaction time. Since we dropped the second selective attention task (flanker task), we decided to include two measures from the visual search task to allow for CFA models to have two measures for each process.

#### 2.4.4. Flanker task

The flanker task was adapted from Rueda et al. (2004) and assessed children’s selective attention (Breckenridge et al., 2013; Senzaki et al., 2018). This task was excluded from analyses due to insufficient data; see Supplementary material for further information.

## 3. Results

### 3.1. Descriptive statistics and correlations

Table 2 provides a summary of means, standard deviations, ranges, skewness, and kurtosis for all EF and AC measures. There was neither a floor nor ceiling effect for these tasks, which is often a problem when testing children from 3 to 5 years of age. Table 3 summarizes the inter-correlations between all EF and AC measures (see Supplementary material for confidence intervals). As can be seen, most of the measures were significantly correlated, although the correlations were generally moderate (most ranging between 0.06 and 0.39). Moreover, the pattern of these correlations was not clearly consistent with EF and AC variables demonstrating either a unitary or fractionated model based on the convergent and discriminant validity of the results.

**Table 2:** Mean, standard deviation (SD), range, skewness, and kurtosis for executive function and attentional control measures.

Task	Measure	Mean (SD)	Range	Skewness	Kurtosis
Circle/Triangle	Proportion of correct responses	0.61 (0.32)	0.00–1.00	−0.66	−0.80
Wrapped gift	Composite of latency to first touch and first peek (sec)	374 (129)	17–480	−1.02	0.04
Spin the pots	Proportion of correct searches	0.64 (0.25)	0.00–1.00	−0.48	−0.47
Digit span	Proportion of correct responses	0.57 (0.11)	0.19–0.91	0.08	1.70
Low-frequency CPT	d-prime	3.35 (1.35)	0.36–7.44	−0.20	−0.13
High frequency CPT	d-prime	2.01 (1.23)	−1.76–5.68	0.65	1.11
Fruit visual search accuracy	Proportion of correct searches	0.70 (0.22)	0.13–1.00	−0.82	−0.20
Fruit visual search RT	Average reaction time on correct trials (ms)	4,853 (752)	2,967–7,283	0.26	0.24

**Table 3:** Correlation matrix of executive function and attentional control measures.

	CT	WG	StP	DS	LCP	HCP	VSA	VSR
CT	—							
WG	0.18^*^	—						
StP	0.33^***^	0.19^*^	—					
DS	0.28^**^	0.19^*^	0.36^***^	—				
LCP	0.15	0.25^**^	0.29^***^	0.33^***^	—			
HCP	0.06	0.23^*^	0.23^**^	0.09	0.39^***^	—		
VSA	0.11	0.27^**^	0.38^***^	0.22^*^	0.56^***^	0.30^***^	—	
VSR	−0.33^***^	−0.20^*^	−0.32^***^	−0.29^**^	−0.45^***^	−0.32^***^	−0.41^***^	—
Age	0.43^***^	0.13	0.39^***^	0.36^***^	0.28^**^	0.10	0.21^*^	−0.36^***^

Correlations with age appear on the last line. CT, Circle/Triangle; WG, wrapped gift; StP, spin the pots; DS, digit span; LCP, low-frequency continuous performance task; HCP, high frequency continuous performance task; VSA, visual search accuracy; VSR, visual search reaction time. ^*^p < 0.05, ^**^p < 0.01, and ^***^p < 0.001.

As can be seen in the last row of the correlation matrix in Table 3, children’s performance on all except two of the measures (wrapped gift and high-frequency CPT) improved with age. It should also be noted that children who responded faster on the selective attention task (visual search) were also more accurate (*r*(130) = −0.41, *p* < 0.001), which thus precludes the possibility of a speed-accuracy trade-off.

### 3.2. Confirmatory factor analysis

Confirmatory Factor Analyses (CFA) were conducted to test whether a one-factor model, a two-factor model (EF and AC), or a three-factor model (response inhibition, working memory, AC) fit the data the best. CFAs were run in R using the Lavaan package (Rosseel, 2012). A good model fit was determined using the following statistics: chi-square test with non-significant values, root mean square error of approximation (RMSEA) with values less than 0.08, standardized root-mean square residual (SRSM) with values less than 0.05, Tucker-Lewis index (TLI), with values greater than 0.90, and the comparative fit index (CFI) with values greater than 0.95 (Kline, 2011; Schumacker and Lomax, 2016). Since the models were nested, chi-square difference tests were conducted to compare which of the three models fit the data best. If two models do not differ significantly, then the simpler model is chosen due to its being more parsimonious (Bollen, 1989).

Critically, the models were also compared using the Akaike information criterion (AIC) which evaluates the best model not only in terms of its predictability but also in terms of the number of variables such that more complex models will not always constitute a better fit (Akaike, 1987). Lower AIC values indicate better model fit (Kline, 2011; Schumacker and Lomax, 2016).

**Figure 1:**
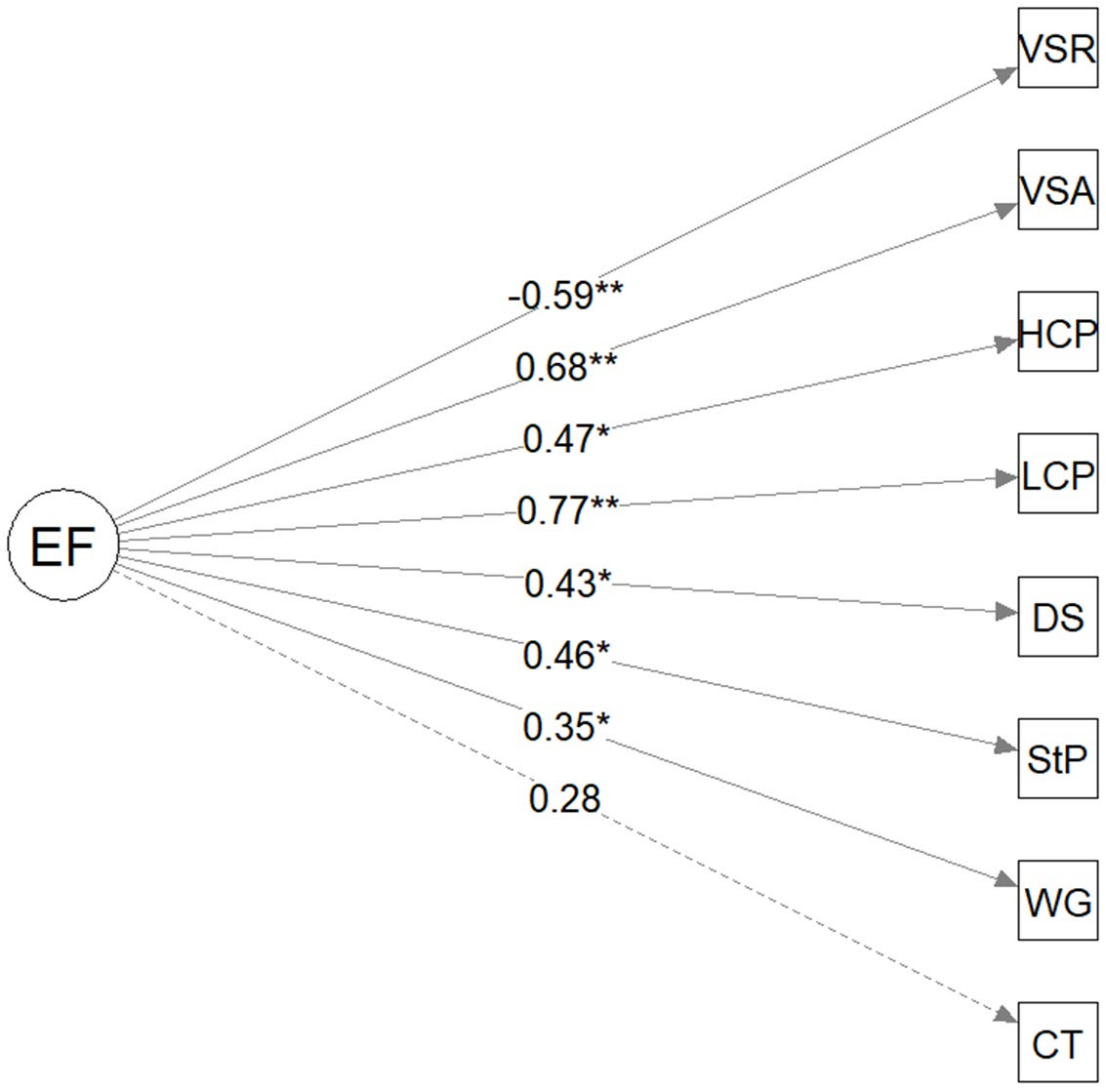
Model path diagram for executive function unitary one-factor model. EF, executive function; CT, Circle/Triangle; WG, wrapped gift; StP, spin the pots; DS, digit span; LCP, low-frequency continuous performance task; HCP, high-frequency continuous performance task; VSA, visual search accuracy; VSR, visual search reaction time. Standard factor loadings and coefficients are shown; ^*^p < 0.05, ^**^p < 0.01, and ^***^p < 0.001.

**Figure 2:**
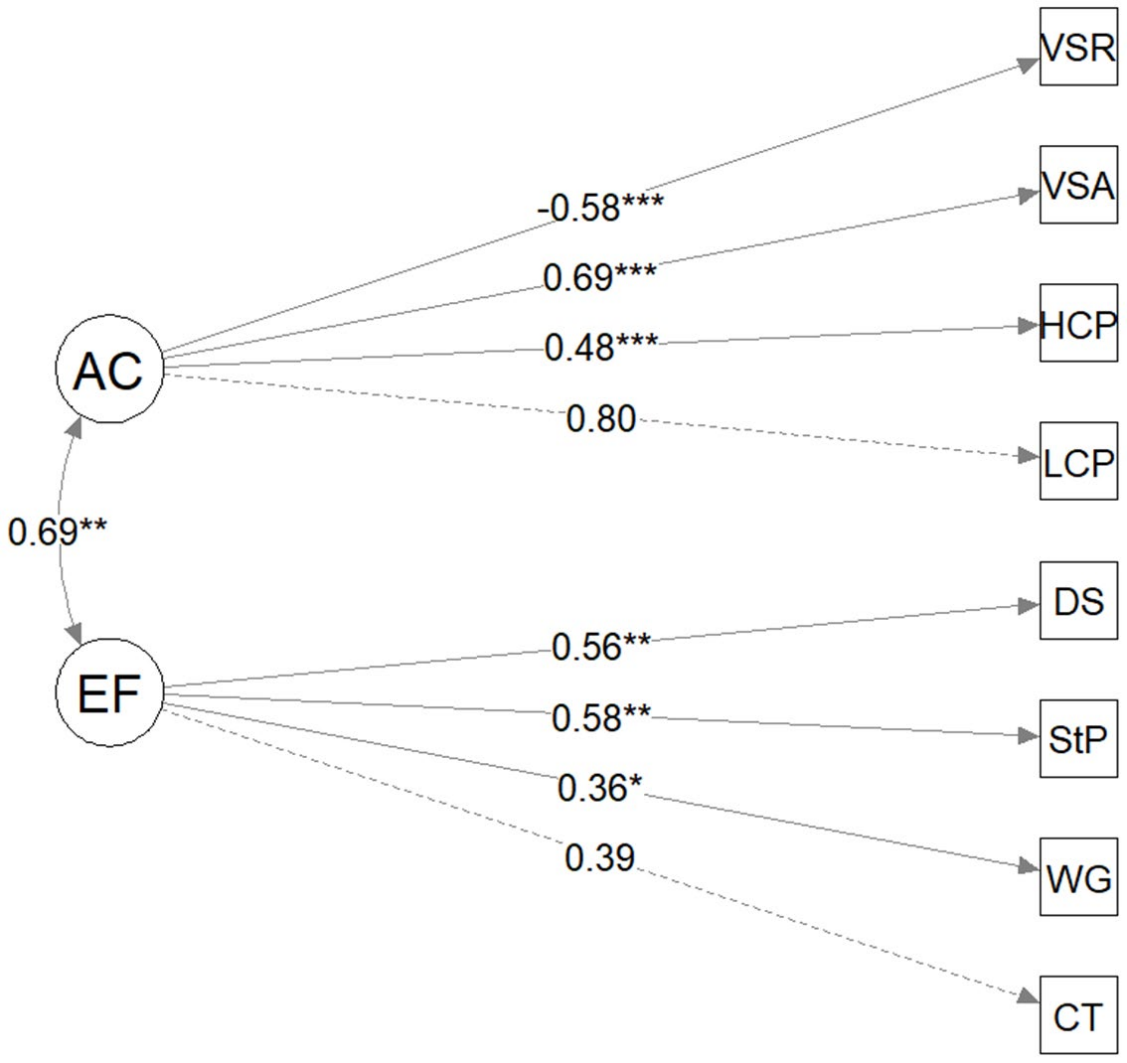
Model path diagram for two-factor model (executive function, attentional control). EF, executive function; AC, attentional control; CT, Circle/Triangle; WG, wrapped gift; StP, spin the pots; DS, digit span; LCP, low-frequency continuous performance task; HCP, high-frequency continuous performance task; VSA, visual search accuracy; VSR, visual search reaction time. Standard factor loadings and coefficients are shown; ^*^p < 0.05, ^**^p < 0.01, and ^***^p < 0.001.

The structure of the unitary one-factor model is presented in Figure 1 and the two-factor model (EF and AC) is presented in Figure 2. Table 4 provides a summary of fit statistics for the one-factor and two-factor model. While some fit statistics indicated that the one-factor model fit the data adequately (i.e., non-significant chi-squared test, RMSEA was 0.06, TLI was 0.90), other fit statistics did not (i.e., SRSM was 0.06, CFI was 0.93). By contrast, all the fit statistics indicate that the two-factor model is a good fit: the chi square was non-significant, the RMSEA was 0.04, SMSR was 0.05, the TLI was 0.97, and the CFI was 0.98. The chi-square difference test indicated that the two-factor model fit the data significantly better than the one-factor model (*x*^2^(1) = 8.64, *p* < 0.001). The AIC was lower for the two-factor model (3946.84) compared to the one-factor model (3953.48). Therefore, the fit statistics and model comparisons indicate that the two-factor model consisting of EF and AC is preferable to the one-factor model. It is nevertheless worth noting that the EF and AC factors are correlated (Figure 2), suggesting that these two factors are related but separable in the current preschool sample.

**Table 4:** Fit statistics for one-factor (executive function), two-factor (executive function and attentional control), and three-factor (response inhibition, working memory, and attention control) models based on confirmatory factor analyses; preferred model is italicized.

Model	*x*^2^ (value of *p*)^a^	df	RMSEA^b^	SRSM^c^	TLI^d^	CFI^e^	AIC^f^	Model comparison	*x*^2^ difference (*p* value)	df difference
Unitary (1)	30.67 (*p* = 0.06)	20	0.06	0.06	0.90	0.93	3953.48			
EF and AC (2)	22.03 (*p* = 0.28)	19	0.04	0.05	0.97	0.98	3946.84	Model 1 vs. *Model 2*	8.64 (*p* < 0.001)	1
RI, WM, AC (3)	21.95 (*p* = 0.19)	17	0.05	0.05	0.95	0.97	3950.76	*Model 2* vs. Model 3	0.08 (*p* < 0.96)	1

a Chi-square test; nonsignificant value of *p* indicates good fit.

b Root mean square error of approximation; values less than 0.08 indicate good fit.

c Standardized root-mean square residual; values less than 0.05 indicate good fit.

d Tucker-Lewis index; values greater than 0.90 indicate good fit.

e Comparative fit index; values greater than 0.95 indicate good fit.

f Akaike information criterion; lower values indicate better fit when comparing models.

**Figure 3:**
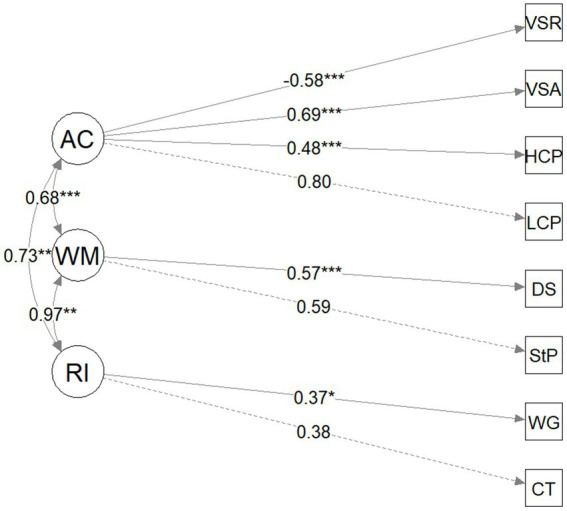
Model path diagram for three-factor model (response inhibition, working memory, attentional control). RI, response inhibition, working memory; AC, attentional control; CT, Circle/Triangle; WG, wrapped gift; StP, spin the pots; DS, digit span; LCP, low-frequency continuous performance task; HCP, high-frequency continuous performance task; VSA, visual search accuracy; VSR, visual search reaction time. Standard factor loadings and coefficients are shown; ^*^p < 0.05, ^**^p < 0.01, and ^***^p < 0.001.

The three-factor model (response inhibition, working memory, and AC; see Figure 3) fit statistics indicated that it fit the data adequately (see Table 4): the chi-square test was not significant, RMSEA was 0.05, SMSR was 0.05, the TLI was 0.95, and the CFI was 0.97. Critically, however, the chi-square difference test indicated that the three-factor model did not fit the data significantly better than the two-factor model (*x*^2^(1) = 0.08, *p* = 0.96). Therefore, the simpler two-factor model was preferred (Bollen, 1989). Further, the AIC was lower for the two-factor model (3946.84) compared to the three-factor model (3950.76). It is worth noting that the response inhibition and working memory are almost perfectly correlated (Figure 3), further suggesting these two factors likely reflect the same underlying process. In summary, the fit statistics, model comparisons and factor correlations collectively indicate that the two-factor model consisting of EF and AC is the preferred model for the current preschool sample.

## 4. Discussion

The primary aim of the current study was to examine whether EF and AC are best characterized as a unitary or multi-factor model for children between 3- and 5 years of age. Most prior studies examining the structure of EF during the preschool years conclude that EF conforms to a unitary and undifferentiated factor model (e.g., Wiebe et al., 2008). AC is assumed to be implicit in all EF processes (Garon et al., 2008), but the current results suggest that further testing is needed before concluding a unitary model. We tested three different models with children between 3.5 to 5 years of age, and the results revealed that EF and AC were separable dimensions in a two-factor model and that response inhibition and working memory were not separable dimensions.

Critically, these results challenge the prevailing view that EF is best conceptualized as a unitary construct during the preschool period, but they also do not support the opposing view that has appeared in the literature. Prior studies indicating that EF is a multi-dimensional construct typically report that response inhibition and working memory represent different factors (e.g., Lerner and Lonigan, 2014). By contrast, this study suggests that EF and AC represent two separable factors. We also tested a three-factor model that differentiated response inhibition and working memory into separate factors, but this three-factor model did not constitute a better fit of the data. Although this latter finding is consistent with previous results suggesting that response inhibition and working memory are not separable processes during the preschool years, our results clearly do not imply that EF itself is a unitary construct. Instead, the current results demonstrate that AC is separable from EF, and therefore suggests that it is important to include direct assessments of AC to fully test the structure of EF during this period of development.

What are the implications of these findings? First, attention is not fully integrated with other EF processes for preschool children, and we cannot simply assume that attention is common to all EF processes. It is instead important to include direct assessments of attention to examine how it influences EF development throughout early childhood. Second, AC processes continue to develop during the preschool years (Ruff and Rothbart, 2001), which will differentially influence children’s performance on EF tasks with different attentional demands. These points are especially important when considering EF as an indicator of children’s school readiness and academic performance (e.g., Micalizzi et al., 2019; Nguyen and Duncan, 2019) because the current findings suggest that a complete assessment of school readiness should include measures of both AC as well as EF.

It is also important to appreciate that there are multiple AC processes (e.g., sustained attention, selective attention) that are associated with different EF subdomains, such as the allocation of attention toward different representations in memory or shifting attention to inhibit a pre-potent response (Putnam et al., 2002). As such, there is no one-to-one relation between EF and AC, because there are multiple modes of operation within each of these attentional systems (Awh et al., 2006). Distinguishing between different models of EF is partially dependent on the focus of the study and chosen analytic method (Miller et al., 2012). Whereas studies examining the associations between specific AC and EF subdomains highlights the commonality between the two (e.g., Espy and Bull, 2005), studies examining the underlying structure may highlight the distinctiveness of EF and AC (e.g., Allan et al., 2015). As such, seemingly contradictory conceptualizations regarding the structure of EF and AC may simply be a function of studies focusing on different questions.

Aside from questions concerning the structure of AC and EF, it is important to appreciate that the ability to successfully plan and complete typical activities most likely depends on the ability to efficiently coordinate multiple EF and AC processes. For example, a child needs to first select and then sustain attention on a book or movie before encoding the plot and characters in working memory. Likewise, children may need to keep homework instructions in working memory and simultaneously inhibit the desire to partake in a more desirable activity (e.g., video games) to sustain attention long enough to complete their math homework. The key may not be to simply perform well on EF and AC subdomains and tasks in isolation, but to be able to flexibly coordinate different subdomains as the context changes (Garon et al., 2008).

We focused our study on specific EF processes (response inhibition, working memory) and AC processes (sustained attention and selective attention), but it is worth noting that we excluded other EF and AC processes found in the preschool literature. Specifically, the current study did not include executive attention proposed by Posner and colleagues or set shifting, sometimes referred to as cognitive flexibility, from the EF literature. It was key to our study to include more than one task per process; we were concerned that including any more tasks would fatigue preschool children’s patience and potentially compromise their performance. We decided not to include executive attention and set shifting since tasks assessing these processes during the preschool years are functionally very similar to each other, as well as to “cool” response inhibition tasks. As one example, Breckenridge et al. (2013) assessed executive attention using a task adapted from the Day/Night task (as we did for our “cool” response inhibition task). They also included an adapted version of the Wisconsin card sorting task, which is often used in the preschool literature to assess set shifting/cognitive flexibility and requires children to inhibit an old rule in favor of following a new rule which is akin to other “cool” response inhibition tasks (Diamond, 2013; Doebel and Zelazo, 2015). As another example, the flanker task can also be considered an index of executive attention (Ruff and Rothbart, 2001) and cognitive flexibility (Griffin et al., 2016). We therefore decided to focus on EF and AC processes and tasks with less overlap to more directly test how attention factored into the underlying structure of EF in preschool children.

Although we have focused thus far on the results derived from the confirmatory factor analysis, a few of the correlational results merit further discussion. First, children’s performance on all but two tasks improved with age, confirming that both EF and AC are developing during the preschool years (Ruff and Rothbart, 2001; Garon et al., 2008; Griffin et al., 2016). Second, there is some debate as to whether “cool” and “hot” response inhibition represent a unitary or separable processes (Sulik et al., 2010; Allan and Lonigan, 2011). Performance on the “cool” circle/triangle task did improve with children’s age, while performance on the “hot” wrapped gift did not. These results suggest that the two exhibit different developmental trajectories (Zelazo and Carlson, 2012). Still, there was also a very modest, but significant correlation between the “cool” circle/triangle and “hot” wrapped gift, suggesting that the two are not entirely independent. Therefore, the results suggest that performance on “hot” and “cool” response inhibition tasks are related, but nevertheless they are sufficiently separable that they follow different developmental trajectories (Hongwanishkul et al., 2005; Willoughby et al., 2011).

### 4.1. Limitations and future directions

While the current study offers new and important insight into how EF and AC are related during the preschool years, there remain a few caveats. First, this pattern of results was based on a sample drawn primarily from middle socio-economic status (SES) families. Prior results indicate that SES interacts with both EF and AC in early childhood (e.g., Watts et al., 2018) and that children from different backgrounds may exhibit different patterns of relations between EF and AC subdomains (Chang and Burns, 2005; Lan et al., 2011). Second, the size of the sample precluded our ability to classify children into different age groups to test whether the structure of the EF and AC subdomains may also develop and change during the preschool years (e.g., Breckenridge et al., 2013). Third, the exclusion of the second selective attention task (flanker task) compromised our ability to reliably test whether selective and sustained attention processes represent one factor or two in preschool children (Hrabok et al., 2007; Steele et al., 2012; Breckenridge et al., 2013). As we discussed in the introduction, it is not possible to distinguish between task and construct factors when the results are limited to one task. Lastly, we included only one “hot” and one “cool” response inhibition tasks in our protocol, and thus any suggestions regarding the unitary vs. separable structure of these tasks are tentative at best. It will be important for future studies to examine how multiple EF and AC subdomains and tasks are related in a larger, more diverse sample of preschool children. It also remains to be seen whether EF and AC become more separable or integrated in children beyond the preschool period.

## 5. Conclusion

Attention is central to the emergence and development of EF during early childhood (Garon et al., 2008), but direct assessments of AC are not usually included when examining the underlying structure of EF in preschool children. We did so in the current study and found that AC is separable from EF subdomains in children from three to five years of age. This study thus highlights the importance of including direct assessments of multiple AC subdomains to investigate and understand how the structure of EF changes during the preschool years. The implications of these findings are important for better understanding the development of executive function and attentional control, as well as providing new insights into how variations in EF and AC may operate in developmental disorders (e.g., Otterman et al., 2019).

## Data availability statement

The datasets presented in this study can be found in online repositories. The names of the repository/repositories and accession number(s) can be found at: Open Science Framework, https://osf.io/8wxt9/ (DOI: 10.17605/OSF.IO/8WXT9).

## Ethics statement

The studies involving human participants were reviewed and approved by Indiana University Institutional Review Board (IRB). Written informed consent to participate in this study was provided by the participants’ legal guardian/next of kin.

## Author contributions

AD and BB were both responsible for the conceptualization and design of the study. AD completed data collection and all statistical analyses. AD wrote the manuscript and BB provided critical oversight and feedback. All authors contributed to the article and approved the submitted version.

## Funding

This research was supported with funding from the US Army Research, Development and Engineering Command Acquisition Center (W911NF-13-2-0045).

## Acknowledgments

Portions of these data were previously presented at the biennial meetings of the Society for Research on Child Development (SRCD), Baltimore MD, 2019 and the Annual Meeting of the Cognitive Science Society, Toronto, Canada, 2022. This study was also part of the first author’s dissertation. The authors would like to thank the children and parents who participated, and Jennifer Meyer, Allison Higgs, Adefolarin (Fola) Alade, and Oyun-Erdene Chingis for assistance with participant recruitment, stimulus creation, and data scoring.

## Conflict of interest

The authors declare that the research was conducted in the absence of any commercial or financial relationships that could be construed as a potential conflict of interest.

## Publisher’s note

All claims expressed in this article are solely those of the authors and do not necessarily represent those of their affiliated organizations, or those of the publisher, the editors and the reviewers. Any product that may be evaluated in this article, or claim that may be made by its manufacturer, is not guaranteed or endorsed by the publisher.

## Supplementary material

The Supplementary material for this article can be found online at: https://www.frontiersin.org/articles/10.3389/fpsyg.2023.1146101/full#supplementary-material
