# Supplementary material for: How attention factors into executive function in preschool children
Source: Front Psychol. 2023 Jul 12;14:1146101. doi: 10.3389/fpsyg.2023.1146101 (PMC10369189; doi:10.3389/fpsyg.2023.1146101)
Supplement: Supplementary file 1 [file Table_1.pdf]

Supplementary Material

How Attention Factors into Executive Function in Preschool Children

Aditi V. Deodhar* and Bennett I. Bertenthal

***Correspondence:**
Aditi V. Deodhar
aditi.v.deodhar@gmail.com

The flanker task was adapted from Rueda et al. (2004) and assessed children's selective attention (Breckenridge, Braddick, & Atkinson, 2013; Senzaki, Wiebe, Masuda, & Shimizu, 2018). Children were presented with a centrally located green fish facing left or right, flanked by two yellow fishes facing in the same direction (congruent trials) or in the opposite direction (incongruent trials) on an iPad or touchscreen laptop controlled with the Paradigm Experimenter software. After 750 milliseconds, fish food appeared to the left and right sides of the three fish located in the middle of the screen. Children were instructed to help the green fish get breakfast (or a snack) by touching the fish food in the direction the green fish was facing; they had up to 30 seconds to touch the fish food. There were eight practice trials to ensure children learned the task and 48 test trials (24 congruent and 24 incongruent trials); the inter-trial-interval (ITI) was three seconds.

Children's accuracy for both congruent trials and incongruent trials was calculated as the proportion of trials where they correctly touched the food located in the direction the green fish was facing, regardless of the direction the flanking yellow fish were facing. The outcome measure was the difference between the proportion of accurate congruent trials and the proportion of accurate incongruent trials. If children are generally able to selectively pay attention to the central fish (and not the flankers), then they should have similar accuracy scores on congruent and incongruent trials; smaller difference scores are evidence of better selective attention (Posner, 2012; Rueda et al., 2004). For instance, a child who is successully able to selectively focus on the central fish would demonstrate high accuray on congruent trials (e.g., 0.95) as well as on incongruent trials (e.g., 0.93). The difference between these two conditions is very small (e.g., 0.02). By contrast, a child who has diffuclty selectively focusing on the central fish and whose attention is pulled to the flanker fish would exhbiit a different pattern of results. This child would still demonstrate high accuracy on congruent trials (e.g., 0.95) , but score much lower on incongruent trials where the flanking fish are facing the oppsite direction (e.g., 0.50); this would result in a larger difference score (e.g., 0.95 – 0.50 = 0.45).

Contrary to our pilot testing, the majority of children had difficulty completing this task. As seen in Supplementary Table 1, 48 children completed zero trials and 31 children completed less than 50% of trials. The criterion for inclusion in the study was completion of at least 50% of the trials. Reasons for the high attrition included children's refusal to continue after the eight practice trials, children quickly becoming disinterested in the task, or children not following task instructions. In order to be included in a confirmatory factor analysis, it is recommended that each measure should have at least one hundred observations (Schumacker & Lomax, 2016).

Given the difficulties associated with children completing this task, the flanker task was excluded from further analyses.

The mean scores and standard deviations of children who completed at least 50% of the trials were calculated for congruent and incongruent trials; 58 children were included in this summary. Children performed better on congruent (M=0.93, SD=0.14) than incongruent trials (M=0.83, SD=0.28); the congruent-incongruent difference score was (M=0.10, SD=0.26).

Supplementary Table 1
Flanker Task Trials Completed

**Age Range**	**Completed 0 trials (Excluded)**	**Completed less than 50% of trials (Excluded)**	**Completed more than 50% of trials (Included)**	**Total**
41-48 months	26	13	10	49
48-54 months	10	13	24	47
54-60 months	12	5	24	41
Total	48	31	58	137
