# Supplementary material for: How attention factors into executive function in preschool children
Source: Front Psychol. 2023 Jul 12;14:1146101. doi: 10.3389/fpsyg.2023.1146101 (PMC10369189; doi:10.3389/fpsyg.2023.1146101)
Supplement: Supplementary file 2 [file Table_2.pdf]

Supplementary Material

How Attention Factors into Executive Function in Preschool Children

Aditi V. Deodhar* **and Bennett I. Bertenthal**

***Correspondence:**
Aditi V. Deodhar
aditi.v.deodhar@gmail.com

Supplementary Table 2
Correlation Matrix of Executive Function and Attentional Control Measures with Confidence Intervals

	CT	WG	StP	DS	LCP	HCP	VSA	VSR
**CT**	—							
**WG**	0.18*<br>[0.01,0.34]	—						
**StP**	0.33***<br>[0.17,0.47]	0.19*<br>[0.03,0.35]	—					
**DS**	0.28**<br>[0.11,0.43]	0.19*<br>[0.02,0.35]	0.36***<br>[0.21,0.50]	—				
**LCP**	0.15<br>[-0.02,0.32]	0.25**<br>[0.08,0.41]	0.29***<br>[0.12,0.44]	0.33***<br>[0.17,0.48]	—			
**HCP**	0.06<br>[-0.12,-0.23]	0.23*<br>[0.05,0.38]	0.23**<br>[0.07,0.39]	0.09<br>[-0.09,0.26]	0.39***<br>[0.23,0.53]	—		
**VSA**	0.11<br>[-0.07,0.28]	0.27**<br>[0.10,0.42]	0.38***<br>[0.23,0.52]	0.22*<br>[0.05,0.38]	0.56***<br>[0.43,0.67]	0.30***<br>[0.14,0.45]	—	
**VSR**	-0.33***<br>[-0.47,-0.16]	-0.20*<br>[-0.36,-0.03]	-0.32***<br>[-0.47,-0.16]	-0.29**<br>[-0.44,-0.12]	-0.45***<br>[-0.58,-0.30]	-0.32***<br>[-0.47,-0.16]	-0.41***<br>[-0.54,-0.26]	—
**Age**	0.43***<br>[0.28, 0.56]	0.13<br>[-0.04, 0.30]	0.39***<br>[0.23,0.52]	0.36***<br>[0.21,0.50]	0.28**<br>[0.12,0.43]	0.10<br>[-0.07-0.27]	0.21*<br>[0.04,0.37]	-0.36***<br>[-0.50,0.20]

Correlations with Age appear on the last line. CT=Circle/Triangle, WG=Wrapped Gift, StP=Spin the Pots, DS=Digit Span, LCP= Low-Frequency Continuous Performance Task, HCP=High Frequency Continuous Performance Task, VSA=Visual Search Accuracy, VSR=Visual Search Reaction Time. 95% Confidence Intervals are in square brackets
p* < .05, ** *p* <.01, * *p* <.001
